# Treatment selection and treatment initialization in guideline-based stepped and collaborative care for depression

**DOI:** 10.1371/journal.pone.0208882

**Published:** 2018-12-26

**Authors:** Daniela Heddaeus, Maya Steinmann, Anne Daubmann, Martin Härter, Birgit Watzke

**Affiliations:** 1 Department of Medical Psychology, University Medical Center Hamburg-Eppendorf, Hamburg, Germany; 2 Department of Medical Biometry and Epidemiology, University Medical Center Hamburg-Eppendorf, Hamburg, Germany; 3 Clinical Psychology and Psychotherapy Research, Institute of Psychology, University of Zurich, Zurich, Switzerland; Rijksinstituut voor Volksgezondheid en Milieu, NETHERLANDS

## Abstract

In order to optimize patient allocation, guidelines recommend stepped and collaborative care models (SCM) including low-intensity treatments. The aim of this study is to investigate the implementation of guideline-adherent treatments in a SCM for depression in routine care. We analyzed care provider documentation data of n = 569 patients treated within a SCM. Rates of guideline-adherent treatment selections and initializations as well as accordance between selected and initialized treatment were evaluated for patients with mild, moderate and severe depression. Guideline-adherent treatment selection and initialization was highest for mild depression (91% resp. 85%). For moderate depression, guideline-adherent treatments were selected in 68% and applied in 54% of cases. Guideline adherence was lowest for severe depression (59% resp. 19%). In a multiple mixed logistic regression model a significant interaction between guideline adherence in treatment selection/initialization and severity degree was found. The differences between treatment selection and initialization were significant for moderate (OR: 1.8 [95% CI: 1.30 to 2.59; p = 0.0006]) and severe depression (OR: 6.9; [95% CI: 4.24 to 11.25; p < .0001] but not for mild depression (OR = 1.8, [95%-CI: 0.68 to 4.56; p = 0.2426]). Accordance between selected and initialized treatment was highest for mild and lowest for severe depression. We conclude that SCMs potentially improve care for mild depression and guideline adherence of treatment selections. Guideline adherence of treatment initialization and accordance between treatment selection and initialization varies with depression severity. Deficits in treating severe depression adequately may be more a problem of failed treatment initializations than of inadequate treatment selections.

## Introduction

Depression is a widespread disease and causes severe impairments, a high degree of personal suffering [1] and high direct and indirect costs [2]. Detailed estimations predict that the depression-related disease burden will increase in the next 20 years and that depression will represent the most important factor for impairment in high income countries [1]. With a 1-year prevalence of 7.7%, unipolar depression is one of the most widespread mental diseases in the European population [3, 4].

In order to provide effective care for depression, guidelines for evidence-based diagnosis and treatment of unipolar depression have been developed worldwide [5–9]. Recommendations include–among others–screening high-risk groups for depression, distinguishing between different severity levels, providing active monitoring, acute and maintenance therapy as well as carefully assessing self-harm or suicide risk. Some of the recommendations are specific to the disease course or certain patient groups (e.g. chronic course, patients with somatic comorbidities), while most of the treatment recommendations are related to depression severity [5, 6, 9]: For first episodes of mild depression, guidelines recommend active monitoring. Other guideline-adherent treatments for mild depression comprise low-intensity treatments like bibliotherapy, individual guided self-help based on cognitive-behavioral principles, computerized cognitive-behavioral therapy, and structured physical activity programs, as well as psychotherapeutic approaches. Treatment with psychotherapy or pharmacotherapy is recommended for moderately depressed patients, while a combination of psycho- and pharmacotherapy should be provided for severe depression. Next to the criterion of depression severity, guidelines recommend the explicit consideration of patient preferences regarding a shared decision.

While the development of guidelines represents an important improvement, the implementation of their evidence-based recommendations is still insufficient [10–21]. Depression often remains undetected and is diagnosed with delay [22–25], and diagnoses are often unspecific: For instance, analyses of German health insurance data show that 50% of all depression diagnoses do not specify severity degrees according to the “International Statistical Classification of Diseases and Related Health Problems—10”-Classification System (ICD-10) [26]; the majority of these diagnoses were made by general practitioners (GPs) [14]. Partly as a consequence of unspecific diagnostics, treatment selection is often also not specific and thus not in accordance with the guideline: For instance, according to a British primary care study, GPs prescribe antidepressants to 49% of patients with mild depression [27], although guidelines recommend not using antidepressants routinely to treat mild depression due to their poor risk-benefit ratio [5]. A general challenge is to actually apply and initiate adequate interventions [28, 29]: A European study shows that only 23% of patients with depression or anxiety disorders were provided an adequate treatment [11, 25] while in a Canadian survey only 30% of patients with mood disorders received any treatment at all [30]. Finally, only 50% of patients with moderate to severe depression or dysthymia received guideline-adherent interventions (antidepressants, psychotherapy, or the combination of both) for a sufficiently long duration, indicating high rates of undersupply [14].

Stepped care models as recommended by guidelines may optimize treatment decisions and the provision of evidence-based treatments [31, 32] by allocating as many patients as possible to low-intensity treatments, thus reserving more intensive interventions for patients in greater need [33]. In fixed stepped care, all patients begin with low-intensity interventions and step up if they do not benefit sufficiently. In stratified stepped care, patients are assigned to treatments of different intensities from the start, taking into consideration patient characteristics as well as initial symptom severity. Their treatment course is monitored regularly [33]. Studies addressing the implementation of stepped care vary strongly regarding study population, care model, and comparison groups [34]. Common optional elements are guided self-help, psychoeducation, antidepressant medication and psychological interventions such as cognitive behavioral therapy (low- or high-intensity), brief psychotherapy, interpersonal therapy, motivational interviewing and problem-solving therapy [34].

A guideline-based stepped and collaborative care model (SCM) was implemented in routine care within the research project “Health Network Depression” as part of psychenet–the Hamburg Network for Mental Health [35]. Its aim was the improvement of health care for patients with depression by providing integrated and evidence-based health care according to the German National Clinical Practice Guideline “Unipolar Depression” [32]. A specially trained multi-professional network of GPs, psychotherapists, psychiatrists and in-patient facilities was established. The aim of this network was to optimize diagnostic procedures, to improve treatment selection interventions and thus enable an early detection of depression followed by a prompt, professional and effective and efficient treatment. The SCM integrated various evidence-based treatment options of different intensities, including innovative low-intensity interventions based on cognitive-behavioral methods. This is the first evaluation of the systematic application of these interventions within a SCM in German routine care [36].

Against the background of insufficient guideline implementation [11, 12, 14, 15, 28] and lack of knowledge about the reasons for this shortcoming, it is important to analyze which treatments were selected at first place and whether they were actually initialized [18]. We defined initially selected treatments as treatment recommendations given by the GP under consideration of the preference and motivation of the patient, while we defined actually initialized treatments as the first treatment a patient received in the SCM. This is also highly relevant taking into consideration that the first intervention in a patient’s treatment pathway plays a decisive role in the further care process [33].

The objective of this study was to investigate selection and initialization of first treatments patients receive in a guideline-based SCM for depression in primary care. More specifically, we aimed to investigate the following research questions:

1. How are treatment selection and treatment initialization implemented with regard to guideline-adherence for patients with different severity levels of depression?
2. To what degree do selected and initialized treatments match?

## Methods

### Study design and setting

This study is part of a larger project evaluating the SCM’s effectiveness and cost-effectiveness in a randomized controlled intervention trial of a consecutive sample of depressed patients from primary care, with assessments at baseline and 3, 6 and 12 months after baseline [36]. Through cluster-randomization on the level of the participating primary care units, GPs were divided into two groups: GPs in the intervention group treated patients within the SCM, whereas GPs in the control group carried out treatment as usual (for details and results concerning the main study see [36, 37]).

The analyses provided here refer solely to the intervention group, i.e. to the GPs applying SCM and the patients receiving SCM. A comparable analysis between IG and CG referring to the research described above was not possible to perform, as there is no data available on the CG patients’ diagnoses nor on the treatments selected and initialized by the GPs in the CG which is explained by the study design and main research question of the overall study. Within a cross-sectional approach, we analyzed data documented by health care providers regarding diagnostic procedures, treatment decisions and monitoring routines as well as data from questionnaires completed by patients at baseline and 3 months after baseline. Patient enrollment took place from October 2012 to March 2014.

#### Ethical approval

The study was approved by the responsible local Ethics Committee of the Chamber of Psychotherapists in Hamburg and conducted according to the principles of the Declaration of Helsinki (2013 version). The study protocol was registered under ClinicalTrials.gov, NCT01731717.

#### Patient recruitment

Patient recruitment was carried out by the participating GPs and comprised three guideline-recommended assessment steps supported by checklists [38]: First, the GP systematically screened patients with particular risk factors for depression (presence of diffuse somatic symptoms and/or chronic somatic conditions [32, 39]) by applying a 2-item checklist. If screened positively, the GP assessed the main depression criteria (persistent low mood and/or loss of interest or pleasure) with the 2-item depression screener [40]. If at least one of two criteria was fulfilled, the patient was assessed with the depression module of the Patient Health Questionnaire-9 (PHQ-9, [41]). The study’s inclusion criteria were a score of five or more points on the PHQ-9, informed consent and a minimum age of 18 years. Patients with insufficient knowledge of German or a health situation that did not allow study inclusion were excluded, as well as patients already receiving psychotherapy or pharmacotherapy for a mental disorder on the day of entering the study. Neither use of psychotherapy or pharmacotherapy in the past during a prior episode nor somatic or mental comorbidities were exclusion criteria.

### Stepped and collaborative care network

The stepped and collaborative care intervention implemented in our study is a complex intervention consisting of several guideline-based components such as a multiprofessional network trained in guideline-based diagnostics and treatment of depression, optimized diagnostic procedures, improved indication, specific evidence-based treatment options of different intensities (including innovative low-intensity interventions based on cognitive-behavioral methods), standardized monitoring procedures, regular quality circles and an online platform[36].

A necessary framework to promote collaborative treatment in the SCM is a network consisting of the relevant care providers involved in treating depressive patients, i.e. GPs, psychotherapists, psychiatrists, day care and inpatient care facilities [38, 42]. One of the network’s main aims was to enhance cooperation and communication between network members to increase quality of care. Another important aim was the prompt referral from the GP to secondary care, i.e. to a psychotherapist or psychiatrist. Both aims were supported by an online platform specifically developed for the needs of the network in order to book available treatment capacities in secondary care. To ensure that defined quality standards were met and to promote cooperation and information exchange between participating care providers, quarter-yearly quality circles took place [40, 43]. Additionally a network booklet with personal contact data for use within the network was made available. Personal contact with another network care provider in order to refer a jointly treated patient by phone or mail was rewarded by incentives. Previous to the implementation of the SCM, participating care providers obtained training regarding guideline recommendations for diagnostics and treatment [32], rationale and concept of the SCM. Further incentives were given to the care providers for every diagnostic and treatment activity that was not covered by routine care.

#### Diagnostic process and treatment selection

After screening patients, GPs continued the diagnostic process by determining depression type and severity with a checklist of ICD-10 depression criteria (*“ICD-10-checklist”*). Patients were informed about depression and treatment options. Treatment decisions were made following principles of shared decision-making and documented on the *“treatment decision checklist”*. Treatments were allocated following a stratified approach considering depression severity and patient preferences as recommended by guidelines [5, 32].

#### Treatment options and guideline adherence

Patients were offered one of several treatment options of different treatment intensities [36]. The definition which treatment option was guideline adherent for which type of depression is based on national and international depression guidelines following the criteria of severity degree [5, 6]. The German guideline for depression is similar to the NICE guideline for depression. One difference is that the German guideline refers to depression definitions according to ICD-10 whereas the NICE guideline and the Australian and New Zealand clinical practice guideline for the treatment of depression refer to the definitions according to the DSM-IV. As opposed to the NICE guideline, the German and Australian and New Zealand did not explicitly recommend low-intensity treatments like bibliotherapy, internet-based self-help or self-help groups in the short form of their recommendations. In this study, a treatment was defined as guideline-adherent as follows [5, 6]: for mildly depressed patients, active monitoring, all low-intensity interventions like bibliotherapy [44] or internet-based self-help [45] guided by the GP and telephone-based psychotherapy [46] provided by a licensed psychotherapist, and psychotherapy were considered adequate treatments. For moderate depression, psychotherapy, pharmacotherapy, telephone-based psychotherapy and a combination of a low-intensity treatment combined with either pharmaco- or psychotherapy were considered guideline-adherent. For severely depressed patients, the combination of psychotherapy and pharmacotherapy in an in- or outpatient setting was defined as guideline-adherent. If the selected treatment was bibliotherapy the patient was given a self-help book to work with [44]. For the internet-based self-help treatment the patient received a link and a license for the internet program [45]. Patients with the selection of telephone-based psychotherapy were given a description of the treatment including the telephone number of the telephone psychotherapist who awaited their call [46]. For referring the patient to colleagues for psychotherapy or psychiatric treatment or the combination of both in an outpatient setting, the GP booked the treatment option at a certain care provider who indicated free capacities on the online platform. Following this the GP gave the patient this care provider’s phone number to arrange an appointment. For the selections of an inpatient treatment the GP was supposed to call the responsible contact person in one of the cooperating inpatient care facilities and organize hospitalization.

#### Monitoring

Depression severity was monitored systematically by the responsible care provider (GP, psychiatrist or psychotherapist) within predefined time intervals and procedures.

### Variables and measurement

Assessment of sociodemographic data was part of the baseline questionnaire patients filled out directly after having given informed consent. The depression diagnosis according to ICD-10 was extracted from the ICD-10-checklist in which the GP specified the severity degree. The classification algorithm for severity is derived from the ICD-10[26]. Here symptoms of depression are subdivided into main symptoms and additional symptoms. In case of two main and two additional symptoms the diagnosis is a mild depression. Patients with two main and three to four additional symptoms suffer from a moderate depression. A severe depression is diagnosed, if patients report three main and more than four additional symptoms.

Information about the initial treatment selection was extracted from the treatment decision checklist filled out by the GP.

We defined treatment initialization as the first treatment a patient received up to three months after treatment selection. Initialized treatments were extracted from the monitoring checklists filled out by care providers during monitoring appointments. If no information about an appointment with any care provider during the first three months after treatment decision was available, we used data from the patient questionnaire three months after study inclusion.

Selected and initialized treatment was compared on patient level to determine whether they matched. If selected and carried out treatment were identical or had the same level of intensity (i.e., bibliotherapy or internet-based self-help; pharmaco- or psychotherapy), this was defined as “treatment selection implemented”. If a patient received a treatment of higher intensity than the one originally selected, this was categorized as “more intensive treatment than selected”. If a patient was treated with a less intensive intervention than the one selected or not treated at all, this was defined as “no or less intensive intervention”. Dropouts were defined as cases for which neither care provider data nor follow-up patient questionnaires were available.

### Statistical methods

Data was analyzed descriptively by computing frequencies for categorical data and means and standard deviations for continuous data. Patients were grouped by their initial depression severity. We conducted a multiple mixed logistic regression model with severity degree (mild vs. moderate vs. severe), type of depression (recurrent vs. non-recurrent), status of treatment (TS vs. TI) and all of their 2-way and 3-way interactions as fixed effects and patient as a random effect. We performed a backward selection of the non-significant interactions. Level of significance was set at p<0.05, two-sided. The results are represented with odds-ratios and their 95% confidence intervals and p values. These analyses are carried out with SPSS, Version 23 (IBM Corp., Armonk, NY, USA) or with SAS, Version 9.4 (SAS Institute Inc., Cary, NC, USA.)

## Results

### Sample description

N = 610 patients were included into the study (for details see [37]). During the one-year study period, 41 patients (7%) revoked informed consent due to having found a psychotherapist outside the network or not wishing to fill out any more questionnaires. Therefore, a sample of n = 569 patients was analyzed.

Table 1 shows the baseline characteristics of the analyzed sample. According to the PHQ-9, patients reported moderately severe depressive symptoms on average (M = 15.3; SD = 4.7). 98.9% of patients received a specific ICD-10 diagnosis from their GP. More than half (52%) of the patients were diagnosed with a recurrent depression. Ten patients were screened positively according to the PHQ-9 (≥5 points), but their symptoms were not sufficient to receive an ICD-diagnosis. These patients with subthreshold depression were included into the category of mild depression.

**Table 1:** Baseline characteristics of the analyzed sample (n = 569).

	M (SD) / n (%)	missing data n (%)
age (years)	42.08 (13.46)	1 (0.2%)
sex (female)	412 (72.4%)	0
nationality		55 (9.7%)
German	476 (83.7%)	
other European	27 (4.7%)	
non-European	11 (1.9%)	
currently living in a relationship	308 (54.1%)	53 (9.3%)
education		60 (10.5%)
no studies	12 (2.1%)	
secondary general school_a_	116 (20.4%)	
intermediate secondary school_b_	157 (27.6%)	
university entrance diploma_c_	143 (25.1%)	
university degree	81 (14.2%)	
current work situation		61 (10.7%)
unemployed	148 (26.0%)	
part-time employment	124 (21.8%)	
full-time employment	236 (41.5%)	
depression severity (PHQ-9)	15.29 (4.68)	0
health-related quality of life (SF-12)		69 (12.1%)
psychological scale	28.41 (8.33)	
physical scale	44.64 (10.59)	
chronic disease present	234 (41.1%)	44 (7.7%)
diffuse somatic impairments present	516 (90.5%)	40 (7%)
depression diagnosis according to ICD-10		6 (1.1%)
subthreshold depression	10 (1.8%)	
mild depression	75 (13.2%)	
*proportion recurrent*	47 (8.3%)	
moderate depression	296 (52.0%)	
*proportion recurrent*	149 (26.2%)	
severe depression	182 (32.0%)	
*proportion recurrent*	105 (18.5%)	

_a_German: Hauptschule (9 years of education)

_b_German: Realschule (10 years)

_c_German: Fachhochschulreife (12 years) or Gymnasium (12 to 13 years).

### Treatment selection, initialized treatments and guideline adherence

Table 2 displays the results of the analyses regarding initial treatment selection and initialized treatments (research question 1).

**Table 2:** Initial treatment selection (TS) and actual treatment initialization (TI) in %.

Treatment	Level of depression severity						total (N = 569)	
	mild (n = 85)		moderate (n = 296)		severe (n = 182)			
	TS	TI	TS	TI	TS	TI	TS	TI
active monitoring/GP consultation	**15.3**	**11.8**	5.1	11.5	2.7	12.1	5.8	11.6
bibliotherapy	**40.0**	**38.8**	7.4	7.4	1.6	2.2	10.4	10.4
internet-based self-help	**20.0**	**18.8**	7.1	7.8	0.5	1.6	6.9	7.4
telephone-based psychotherapy	**10.6**	**7.1**	**4.1**	**2.7**	1.1	2.2	4	3.3
psychotherapy	**2.4**	**2.4**	**40.2**	**26.7**	21.4	22.5	28.3	21.6
pharmacotherapy	1.2	2.4	**14.9**	**15.9**	5.5	25.3	9.7	16.9
combined psycho- & pharmaco-therapy	2.4	2.4	6.1	6.8	**53.3**	**18.1**	20.6	9.8
other combination	**2.4**	**5.9**	**8.4**	**8.8**	3.7	5.5	6	7.2
inpatient treatment	0.0	0.0	1	0.0	**6**	**0.5**	2.5	0.2
no intervention	0.0	1.2	0.0	2.0	0.0	0.0	0.0	1.2
missing treatment decision	5.9	-	5.7	-	3.8	0.0	6	0.0
dropout	0.0	9.4	0.0	10.5	0.0	9.9	0.0	10.4
**Proportion of guideline adherence**	**91%**	**85%**	**68%**	**54%**	**59%**	**19%**	**68%**	**47%**

Bold numbers mark guideline-adherent treatment selection and initialization for the specific severity degree.

Numbers in columns “TS” show the percentage of patients who selected the respective treatment in the treatment decision process. Numbers in columns “TI” reflect the percentage of patients for whom the respective treatment was initialized afterwards. These numbers were assessed independently from which treatment was selected before, thus numbers do not allow to conclude which proportion of selected treatments were actually implemented by simple subtraction. For about two thirds of the 85 patients suffering from *mild depression*, bibliotherapy was the most frequently selected treatment (40.0%). It was also the most frequently carried out (38.8%), followed by internet-based self-help (20.0% and 18.8%, respectively). Treatments of higher intensity (psychotherapy, pharmacotherapy, a combination of both) were selected in 6.0% of patients with mild depression and initialized in 7.2%. One patient (1.2%) received no treatment, while no further data was available for 8 patients (9.4%) with mild depression. In sum, the rate of guideline-adherent treatment selection was 91% and the rate of guideline-adherent treatment initialization was 85% for patients with mild depression.

In *moderately depressed* patients, the most frequent treatment selected was outpatient psychotherapy (40.2%). It was carried out as an initial treatment in 26.7% of this patient group. Pharmacotherapy was selected for 14.9% of moderately depressed patients and 15.9% received it. Inpatient treatment was selected for 3 patients (1.0%), but none received this intervention. Telephone-based psychotherapy was selected as initial treatment in 4.1% and actually carried out in 2.7% of moderate depression cases. In sum, 68% of initial treatment selections and 54% of initialized treatments are considered to be guideline-adherent for patients with moderate depression.

For *severely depressed patients*, in more than half of the cases (59.3%) the selected treatment was a combination of psycho- and pharmacotherapy as advised by the guidelines, followed by stand-alone outpatient psychotherapy (21.4%). However, initialization of combined psycho- and pharmacotherapy often did not take place: only 18.6% of severely depressed patients actually received this combination. In accordance with the guidelines, low-intensity interventions were seldom chosen (3.2%) and rarely applied (6.0%). In 6.0% of the cases, an inpatient setting was selected, but only 0.5% (1 patient) actually received it. Pharmacotherapy as a stand-alone treatment was the most frequently applied treatment in patients with severe depression (25.3%). None of the severely depressed patients remained without any active treatment (with the exception of dropouts, see next paragraph). In sum, 59% of initial treatment selections and 19% of initialized treatments can be considered guideline-adherent for patients with severe depression.

Patients from all three severity levels had comparable dropout rates of approximately 10%. The proportion of patients whose initial treatment was a further GP consultation without any specific intervention was also about 11% in all groups.

Summing up the proportions of guideline adherence for the total sample, treatment selections were guideline-adherent in 68% of the cases, and treatments actually initialized were guideline-adherent in 47% of the cases.

The multiple mixed logistic regression revealed a significant interaction between status of treatment (TS vs. TI) and severity degree (mild vs. moderate vs. severe).

Table 3 shows that for patients suffering from moderate depression, the odds of obtaining a guideline adherent treatment selection were 1.8 times higher than for guideline adherence in treatment initialization [95% CI: 1.30 to 2.59; p = 0.0006]. For severely depressed patients the odds for a guideline adherent treatment selection were 6.9 times higher than for guideline adherent treatment initialization [95% CI: 4.24 to 11.25; p < .0001]. Accordingly the difference in guideline adherence between treatment selection and initialization is significant for moderate depression as well as for severe depression but not for mild depression (OR = 1.8, [95%-CI: 0.68 to 4.56; p = 0.2426]). Furthermore, there was no difference in guideline adherent treatment rates between patients with a recurrent depression and those with a first occurrence of depression (OR = 1.1, [95%-CI: 0.81 to 1.50; p = 0.5500]). This effect is the same at treatment selection and treatment initialization.

**Table 3:** Guideline adherence in treatment selection (TS) and initialization (TI).

Difference in guideline adherence referring to treatment selection and treatment initialization							
Severity degree	TS		TI		TS vs TI		
	n	%	n	%	OR	95% CI	p
Mild	77	91	72	85	1.762	0.68–4.56	0.2426
Moderate	200	68	160	54	1.836	1.30–2.59	0.0006
Severe	108	59	34	19	6.903	4.24–11.25	< .0001

### Accordance between selected and initialized treatments

**Fig 1:**
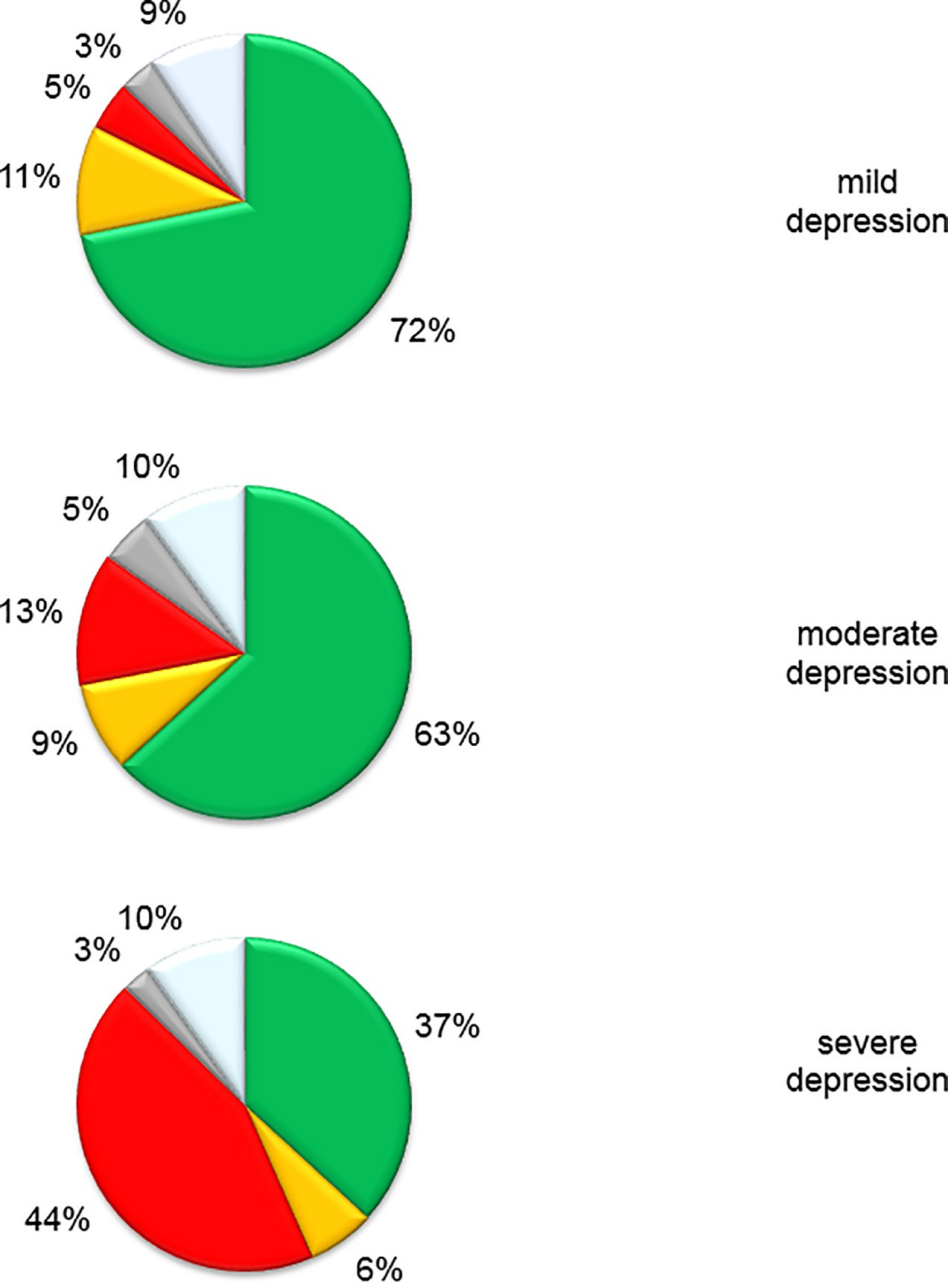
Implementation of treatment decisions. green: treatment decision implemented; orange: treatment implemented more intensive then the selected treatment; red: no or less intensive treatment implemented then the selected treatment; grey: missing treatment decision; white: dropout.

Results for research question 2 underline that the selection of a particular treatment does not automatically lead to its initialization. Fig 1 illustrates the differences between which treatment was selected and which treatment was actually initialized on a patient level. Almost three quarters (72%) of patients with *mild depression* received either the treatment originally decided upon or an equally intensive treatment. Nine patients (11%) received a more intensive treatment than planned and 4 (5%) received a less intensive treatment or no intervention at all. Patients suffering from *moderate depression* received the selected treatment in almost two thirds of cases. The rate of patients who received less intensive treatment than scheduled or none (38 patients; 13%) was somewhat higher for this group than for the group of patients with mild depression. Of patients with *severe depression*, approximately 45% received the planned treatment while nearly the same proportion of patients (43%) received a less intensive treatment than planned. However, as shown in Table 2, no patient with severe depression remained without any treatment.

## Discussion

We described results regarding guideline-adherent treatment selection and initialization for a sample of n = 569 patients within a stepped and collaborative care model (SCM) in routine care. The SCM aimed to optimize diagnostic procedures, treatment decision processes and implementation of guideline-based interventions. To evaluate whether this sample is representative for the German population of persons suffering from depression two studies are available. The distribution of gender in the sample seems to be comparable with twice as many female than male depression patients [14]. Regarding marital status we found comparable data with 54% currently living in a relationship in our study in comparison to 60% in another large epidemiological health care study and for chronic somatic conditions (41% vs. 47%) in the same study [47]. Concerning the distribution of severity degrees in depression a comparison is difficult, as one of the studies [14] revealed that in routine care 50% of the depression diagnoses are unspecific and thus do not allow a statement about the degree of severity of the depression. In our study almost every patient (98.9%) received a specific ICD-10 diagnosis. As we did not collect data of the diagnostic process and the specific diagnoses in the control group we cannot draw a direct comparison between CG and IG. However, we know from a big external administrative study that only half of the registered depression diagnoses are specific, while 50% remain unspecific [14]. This demonstrates that procedures such as training as well as symptom checklists can substantially increase the specificity of GP diagnoses.

### Guideline adherence regarding initial treatment selections

The results regarding treatment selection mostly show a high guideline adherence. This promising result may be attributed to the specific training in guideline recommendations and shared decision-making processes. The implementation of supporting tools such as the treatment decision checklists for GPs probably contributed as well. To our knowledge, no other studies report data on treatment selection or differentiate between selected and initialized treatments as we did. One partially comparable study analyzed the management of depression in UK general practices in relation to severity degree and reported lower rates of guideline-adherent treatment decisions [27]: 49% of mildly depressed patients were prescribed an antidepressant and only few patients with moderate to severe depression were referred to psychological or psychiatric services (1.6% resp. 5.6%)–results which are not in accordance with the guidelines. In our study, the rate of guideline-adherent treatment selection was highest for patients with mild depression, where antidepressant prescriptions were almost nonexistent and low-intensity treatments were selected frequently. Bibliotherapy was selected and initialized approximately twice as frequently as internet-based self-help and four times as often as telephone-based therapy. One reason for this finding could be, that the book for the bibliotherapy could be handed out immediately while the access to the internet-based self-help (lock-in with a license code) or to the telephone-based therapy (calling the telephone psychotherapist) was less immediate. Another reason could be, that working with a book or a companion is more known and intimate than working with internet-based self-help or telephone based therapy. Treatment selection was less guideline-adherent for moderate and severe depression. For moderately depressed patients, psychotherapy was selected almost three times as often as pharmacotherapy, even though the guideline recommends offering the patient psychotherapy and antidepressants as equivalent treatments. This is in agreement with the results of an American primary care survey finding that two thirds of patients preferred counseling over antidepressant treatment [48, 49]. Patient preferences may have been one cause for selecting treatments that were not guideline-adherent: While guidelines place great importance on severity degree, they also explicitly state that patient preferences and characteristics should be taken into consideration.

### Guideline adherence regarding applied first treatments

The overall proportion of 47% of guideline-adherent initialized treatments in this SCM is twice as high as in primary care, according to a European study reporting that only 23% of patients with anxiety and depression received an adequate treatment [11]. This shows the potential of specific SCMs; however, there is still room for further improvement.

With approximately 11%, the proportion of patients receiving a further GP-consultation as first treatment was similar in each of the three severity groups. For some of these patients, active monitoring had actually been scheduled. The others may have consulted their GP due to failed initialization of the originally selected treatments. The rate of patients who dropped out in the sense of not returning to the GP or any other care provider of the network and not answering any follow-up questionnaire was also comparable over all severity groups. For these approximately 10% of patients, it is also likely that the initialization of the selected treatment failed. However, in routine care attrition rates are usually much higher than those found in our study [14] and even in comparison to other SCMs this rate of probably untreated patients is low. Another implementation study of four SCMs in routine care found attrition rates of about 30% [33].

For mildly depressed patients, guideline adherence in treatment initialization was high: there was a very low rate of misapplication of antidepressants in comparison to routine care [14, 27]. Even compared to other stepped-care models, only 5% oversupply with antidepressants is remarkably low. Gidding and colleagues [50] reported almost 30% of non-severely depressed patients being treated with antidepressants. Additionally, only 16% of non-severely depressed patients received minimal interventions (comparable to low-intensity treatments in our SCM), compared to 73% of mildly depressed patients in our study [50]. This indicates that the SCM’s concept of referring patients with mild depression into low-intensity interventions as a first step and thus reserving more intensive treatments for patients with more severe disorders was successfully implemented. It also reflects substantial acceptance of these interventions, as well as their high and prompt availability and practicality. These results are confirmed by another study showing a high acceptance of all three low-intensity interventions from the perspective of the care providers [42].

Moderately depressed patients received less guideline-adherent interventions than mildly depressed patients, but almost three times higher rates of adequate treatments than severely depressed patients. A considerable proportion of moderately depressed patients were not supplied with psychotherapy as planned, indicating that this may be a crucial aspect of impeded treatment initializations. Possible reasons for this finding will be discussed in paragraph “Potential barriers and resources for guideline-adherent care”.

For severely depressed patients, the guideline adherence of initialized treatments was low, the majority of patients received a first treatment of insufficient intensity. However, all patients received some form of active treatment, while 18% of severely depressed patients received no treatment at all in a routine care study [14]. A comparable SCM study [51] reported that 57% of severely depressed patients received adequate treatments. But these different results are difficult to interpret as the group of severely depressed patients in this study consists of moderately and severely depressed patients opposed to severely depressed patients only in our study. Further on there are different definitions of “adequate treatment”: In our study, severe depression was supposed to be treated with a combination of psycho- and pharmacotherapy in line with NICE guideline [5]. In contrast, in Franx and colleagues’ [51] SCM, either psycho- or pharmacotherapy was considered to be a sufficient treatment to start with for severely depressed patients. If we had defined stand-alone psycho- and pharmacotherapy as adequate treatments for severe depression, our SCM would have achieved a rate of 66% of adequately treated severely depressed patients.

Our results indicate that the lower the severity degree is, the higher the probability to receive adequate treatment is. This is also underlined by the result that confirms the significant difference between guideline-adherent treatment selection and initialization for different severity degrees. This finding corresponds to Franx and colleagues’ [51] stepped care study, in which care provision was found to improve for non-severely depressed patients, but not to the same degree as in patients with severe depression.

### Differentiation between selection and initialization

To our knowledge, this is the first study which differentiates between treatment selection and actual treatment initialization in depression care. Our results demonstrate the gap which exists between treatment selection and actual implementation as well as the interaction between “treatment selection/treatment initialization” and “severity degree of depression”. We found that it is by far not enough to improve care providers’ awareness of guideline recommendations. Unfortunately, even when treatments are correctly selected, they are often not implemented afterwards, especially in cases of severe depression.

### Potential barriers and resources for guideline-adherent care

Failure to initialize a treatment may be caused by different barriers impeding the delivery of adequate interventions. Limited access to adequate care for depression is described as a major barrier by different authors. These can be care provider-related barriers such as insufficient knowledge regarding diagnostics and mental disorders in a primary care setting; factors related to the health care system in general such as insufficiently available and integrated resources or long waiting times for therapy; and patient-related aspects such as fear of stigmatization, time constraints or cultural factors [28, 52, 53].

In the SCM, the barrier of insufficient care provider knowledge regarding diagnostics and psychological disorders was addressed by training providers and enhancing professional exchange between psychotherapists, psychiatrists and GPs in regular quality circles. The fact that a greater amount of specific depression diagnoses were made in our study than in routine care shows that we were successful at improving care providers’ knowledge about depression. Additionally, the knowledge about evidence-based and adequate interventions improved, as indicated by the relatively high rate of guideline-adherent treatment selections. In order to overcome barriers related to the health care system in general, we established a care provider network to provide sufficient treatment capacities. We also developed and implemented an online platform to enhance the referral process (cp. section Multi-professional network). This online platform was used frequently and appreciated by the care providers [42]; however, these measures may still have been insufficient to pave the way from GP to secondary care provider in a relevant proportion of cases. Especially initializing a combination of psycho- and pharmacotherapy for patients with severe depression may have been encumbered by an interface problem. Two or even three care providers may be involved in this complex intervention and there may have been difficulties in the referral processes or the patient’s transition from one care provider to another. Another reason may have been a lack of available psychotherapy resources despite their generally higher availability within the SCM compared to routine care. This could be an explanation for the gap between selected and applied stand-alone psychotherapy in moderately depressed patients as well. A further reason for the insufficient initialization of combined psycho- and pharmacotherapy for patients with severe depression might have been that their higher symptom burden affected treatment implementation. For example, for a severely depressed patient, the challenge to call a psychotherapist for an appointment may be higher than for a mildly depressed patient whose symptoms of social withdrawal and hopelessness are lower. Patients in severe crises might also be more ambivalent towards treatments and decide against a selected treatment before even beginning it, duo to doubts and negative emotions. To address patient-related constraints like these, GPs were supported by psycho-educational materials explaining the nature of depression, symptoms and treatment options, amongst others. For patients with time constraints, interventions with high availability, flexibility and practicality were offered, which were meant to be easy to integrate into daily life. Low-intensity treatments can be applied independently of specific appointments and mobility aspects.

One limitation of this study is the lack of validation of the ICD-diagnoses made by the GPs, as no “gold standard” (e.g. structured diagnostic interviews) was additionally applied. This was not possible in the present study due to organizational limitations. The specificity of the diagnoses may have been partially “forced” by the structure of the symptom checklist used. However, GPs perceived the checklist to be a helpful tool for the diagnostic process [42]. Criteria for defining selection and initialization of an intervention as guideline-adherent were based only on the criterion of severity degree. Although severity is one central criterion for guideline-based treatment decisions in depression, the guideline stresses patient preferences as a further important criterion. As data about patients’ preferences (and about the interaction between patient and GP during the treatment decision process) were not collected, we do not know which treatment decisions and initializations were based on this latter criterion.

Strengths of this study are the large sample size of primary care patients and its assessment of information about the crucial phase of treatment initialization, differentiated into treatment selection and actual treatment initialization. We investigated this under routine care conditions and for different subgroups of depressed patients.

## Conclusion

Several important conclusions can be drawn from this study. First of all, SCMs are feasible and capable of improving diagnostic processes, treatment selection, adequate treatment of mildly depressed patients and application of low-intensity interventions in depression care. Furthermore, this study described the important difference of treatment selection and treatment initialization regarding guideline-adherence. Rates of guideline-adherent treatments varied between patient groups of different depression severities. The low rate of guideline-adherent first treatments in severely depressed patients was not primarily a problem of inadequate treatment selection or lack of knowledge, but rather a matter of failed implementation. Further studies should examine which elements of the treatment initialization and implementation process cause difficulties, such as a lack of treatment availability or problems during referral from one care provider to another. Additionally, it will be important to further investigate which barriers and enablers on the level of care provider, health care system and patient characteristics are related to successful implementation, such as patients’ disease course or physical and mental health condition.

## Supporting information

S1 Table Underlying Data for the analysis: T0_data_181130DH.zip. (ZIP)
